# Characterization and Discrimination of the Taste and Aroma of Quinoa Baijiu Using Electronic Tongue, GC Electronic Nose and Comprehensive Two-Dimensional Gas Chromatography Time-of-Flight Mass Spectrometry

**DOI:** 10.3390/foods15213968

**Published:** 2026-11-05

**Authors:** Shengyuan Guo, Wenting Wang, Wenjun Ou, Kaimian Li, Chuan Dong, Fei Gao, Guixing Ren

**Affiliations:** 1 Environmental Science Research Institute, Shanxi University, Taiyuan 030006, China; guoshengyuan217@163.com (S.G.);; 2 School of Life Science, Shanxi University, Taiyuan 030006, China; 3 Tropical Crops Genetic Resources Institute, Chinese Academy of Tropical Agricultural Sciences, Haikou 571101, China

**Keywords:** quinoa baijiu, electronic tongue, electronic nose, gas chromatography-mass spectrometry, taste, volatile compounds

## Abstract

Quinoa Baijiu, brewed with quinoa as a partial or sole raw material, is an emerging distilled spirit whose flavor characteristics remain poorly characterized. This study aimed to characterize and discriminate the taste and aroma of quinoa Baijiu using electronic tongue (E-tongue), gas chromatographic electronic nose (GC E-nose) and headspace solid-phase microextraction coupled with comprehensive two-dimensional gas chromatography time-of-flight mass spectrometry (HS-SPME-GC×GC-TOF-MS). Three quinoa Baijiu samples (quinoa accounting for 20%, 40% and 100% of the raw materials, QJ20, QJ40 and QJ100) were compared with nine commercial Baijiu samples covering light-, strong- and sauce-aroma types. A total of 201 volatile compounds were identified, among which 70 esters predominated (>60% of the total relative content). E-tongue analysis showed that quinoa Baijiu exhibited significantly higher sweetness-associated and lower bitterness-associated sensor responses than the commercial reference samples. These findings reflect instrumental discrimination and should not be equated with human sensory perception. GC E-nose, combined with hierarchical cluster analysis, clearly differentiated the four Baijiu categories and revealed that the instrumental aroma fingerprints of quinoa Baijiu were similar to those of light-aroma Baijiu. Compounds relatively abundant in or unique to quinoa Baijiu—including ethyl undecanoate, ethyl oleate, diethyl azelate, 1,2-decanediol, acacia alcohol, 2-ethyl heptanoic acid, 2,5-dimethyl-4-hexenoic acid, 3-methylenetridecane, 7-hexadecene and caryophyllene—may serve as candidate volatile markers. These results demonstrate that quinoa Baijiu possesses a distinctive and recognizable instrumental flavor profile, providing a chemical basis for its differentiation from traditional Baijiu.

## 1. Introduction

Baijiu is the most popular alcoholic beverage in China, holding a distinctive place in Chinese traditional culture. Currently, the market features a diverse array of Baijiu brands, each exhibiting unique styles and characteristics. The aromatic profiles of these various Baijiu types differ significantly, attributable to the diverse fermented raw grains and brewing technologies employed [1]. Based on their aromatic characteristics, Baijiu can be broadly divided into twelve types, with strong aroma, sauce aroma, and light aroma recognized as the three primary aroma categories due to their widespread appeal [2]. Consequently, the choice of raw materials used in the brewing process is a critical factor in determining the quality and flavor of Baijiu products.

Native to the Andean region of South America, quinoa (*Chenopodium quinoa* Willd.) is one of the few ancient food crops that has not undergone artificial genetic modification and has been extensively cultivated in the alpine regions of China [3]. Quinoa is highly nutritious, containing starch (52.0–69.0%), lipids (2.0–9.5%), protein (13.8–16.5%), dietary fiber (7.0–9.7%), and essential nutrients that meet daily human intake requirements [4]. Regular consumption of quinoa has been associated with the prevention and management of various health conditions, including inflammation, obesity, diabetes, cancer, cardiovascular diseases, and celiac disease [5]. With the rapid development of the social economy and growing consumer interest in diversified and specialty Baijiu products, quinoa Baijiu, as a deep-processed quinoa product, has gained attention in northern China. However, Baijiu is an alcoholic beverage, and the nutritional properties of quinoa grain cannot be directly extrapolated to the final distilled product. This study therefore focuses on the instrumental flavor discrimination and volatile chemical characterization of quinoa Baijiu, rather than its nutritional or health properties. Its market share in northern China continued to expand, making it an increasingly popular choice among consumers. However, as an alcoholic beverage, the unique taste and flavor profile of Baijiu play a crucial role in attracting consumers.

At present, it is common to analyze the flavor components of different types of Baijiu on the market. For strong aroma Baijiu, researchers identified the important aroma active compounds in Wuliangye Baijiu by using aroma extract dilution analysis (AEDA) and gas chromatography-olfactory (GC-O) determination, including ethyl hexanoate, ethyl 3-methylbutyrate, ethyl octanoate, ethyl 2-methylbutyrate, valeric acid, ethyl octanoate, furfural, 4-methylphenol, hexanoic acid, isovaleric acid, 1,1-diethoxyethane [6]. Among the light aroma Baijiu varieties, Shanxi Fenjiu boasted the longest historical lineage. This distinguished Baijiu was enriched with critical aromatic compounds, including ethyl octanoate, ethyl acetate, phenylacetaldehyde, 4-ethyl guaiacol, 3-methyl-1-butanol, 2-methylpropanol, 1-octanol, ethyl phenylacetate, styrene acetate, and β-phenylethanol. These compounds contributed to the core instrumental response patterns of Fenjiu, imparting distinct fruit, floral, sweet, and alcoholic flavors [7]. Maotai is a well-known brand of sauce aroma Baijiu in China. Researchers used GC-O and gas chromatography-mass spectrometry (GC-MS) to identify 79 aroma compounds in Maotai liquor, among which ethyl acetate, ethyl lactate, and acetic acid had the highest concentrations [8]. Further analysis showed that the aroma characteristics and sensory attributes among different types of Baijiu differed greatly [9]. The stylistic characteristics of quinoa Baijiu and the flavor distinctions between quinoa Baijiu and commercial Baijiu have not been deeply explored.

Although there have been many studies on the flavor of traditional Fen-flavor, Luzhou-flavor and Maotai-flavor liquor, the instrumental taste/odor fingerprints and volatile chemical markers of quinoa liquor are still unclear. This study is the first to combine an electronic tongue, a gas chromatography electronic nose and headspace solid-phase microextraction (HS-SPME) with integrated two-dimensional gas chromatography–time-of-flight mass spectrometry (GC×GC-TOF-MS). The quinoa addition ratios were defined as the mass fraction of quinoa in the brewing raw materials, at 20%, 40% and 100%(*w*/*w*), corresponding to QJ20, QJ40 and QJ100. For comparison, commercial baijiu representing the three major aroma profiles—light-aroma (Fenjiu), strong-aroma (Wuliangye) and soy-sauce-aroma (Maotai)—were selected, covering low, medium and high price ranges. These commercial samples served as market references rather than matched controls. The focus is on instrumental discrimination and candidate volatile biomarkers, rather than causal or sensory conclusions. This study provides preliminary information for sample discrimination and for identifying candidate volatile markers, which may serve as a reference for future studies.

## 2. Materials and Methods

### 2.1. Materials

A total of 12 Baijiu samples were included in this study, and their detailed information is described below. The quinoa Baijiu used in this study was brewed by Shanxi Jinbei Quinoa Development Co., Ltd. (Taiyuan, China) with traditional technology. Three quinoa Baijiu samples with 53% ethanol content were brewed using traditional technology, with quinoa accounting for 20% (QJ20), 40% (QJ40), and 100% (QJ100) of the brewing materials (*w*/*w*), respectively. In addition, commercial Baijiu samples representing the three major aroma types and low-, medium-, and high-price market grades were purchased: strong-aroma Wuliangye (LW, MW, HW), sauce-aroma Maotai (LM, MM, HM), and light-aroma Fenjiu (LF, MF, HF). Price grade was used only to cover different market positions and was not considered a measure of aroma intensity, sensory quality, or product quality. No relationship between aroma intensity and price was assessed in this study. These commercial samples were used as market references, not as matched controls produced under identical conditions. All samples were stored at low temperature and away from light during the experiment. In addition, this study did not include 0% quinoa produced under the same fermentation, distillation and storage conditions. Commercial liquor samples were purchased as market reference samples, not matching with quinoa liquor. It should be noted that the sample size of this study (n = 12) is limited. The sample set was designed as a balanced comparison, with three quinoa Baijiu samples differing in quinoa proportion and three commercial products for each of the three major aroma types (nine commercial samples in total), so that each category was represented by multiple independent products rather than a single sample. Nevertheless, the limited sample size may affect the stability of multivariate models, and the results should be regarded as exploratory.

### 2.2. Determination of Taste by E-Tongue

E-tongue sensor response analysis was performed using the ASTREE II E-tongue system (Alpha MOS Company, Toulouse, France), which consists of six electrochemical sensors [10]. Among them, AHS, CTS, ANS, SCS and NMS are sensitive to sour, salty, sweet, bitter and umami tastes respectively, while CPS is universal [11]. Before the test, the internal solution was added into the E-tongue taste sensor and activated for 24 h. Then the standard reference solution, negative film cleaning solution and positive film cleaning solution were used to conduct self-test on the E-tongue system. After the self-test passed within 30 times, the sample was injected. Each Baijiu sample was diluted to 10% ethanol (*v*/*v*), then placed in a constant temperature water bath at 20 °C. Subsequently, 25 mL of the sample was measured in a special beaker for E-tongue and placed into the sensor for detection. The instrument parameters were as follows: delay = 0 s, collection time = 120 s, interval = 1 s, stirring rate = 60 rpm, and acquisition period = 1. After each measurement, the sensor array was rinsed by ultrapure water for 20 s to prepare for the next measurement. For each sample, five analytical measurements were performed. The last three measurements were used as analytical replicates and averaged for further analysis. These replicates assess analytical precision and are not biological or production replicates.

### 2.3. Odor Analysis by GC E-Nose

The volatile compound extracts from different Baijiu samples were tested by using the Heracles Neo electronic nose (Alpha MOS Company, Toulouse, France) [12]. 1 mL sample of Baijiu was introduced into a 20 mL headspace bottle, which was then sealed with a Teflon spacer and placed in an automatic sampler. Each small bottle was stirred at 40 °C (500 rpm) and incubated for 20 min, then the gas accumulated in the headspace was injected into the trap [13]. The carrier gas (hydrogen) was circulated at 1 mL min^−1^ in constant current mode. The injection volume was 250 µL and the injection temperature was 200 °C. The oven temperature was maintained at 60 °C for 2 s, then raised to 270 °C at a rate of 3 °C per second and held for 20 s. The flame ionization detector was maintained at a temperature of 280 °C. Each sample was analyzed five times as analytical replicates to assess method precision. The mean values were used for sample-level comparison.

### 2.4. Extraction of Volatile Compounds

The SPME conditions were adopted from Zhang et al. [14], in which the extraction parameters were optimized by central composite design and response surface methodology for Baijiu volatile analysis. All samples were extracted under strictly identical conditions, so that the relative peak-area ratios are comparable across samples. All samples were diluted to a final concentration of 10% ethanol (*v*/*v*) using ultrapure water. Then 5 mL of diluted baijiu sample and 2 g of sodium chloride were taken into a 20 mL headspace bottle until saturated. For quantitative analysis, an internal standard of 2-octanol (Sigma, Shanghai, China) was utilized. A volume of 20 µL of 2-octanol, diluted 2000 times, was added to the headspace bottle. The mixture was thoroughly shaken, and the bottle was subsequently sealed with a magnetic cap and a PTFE silicone spacer. Samples were extracted using headspace solid phase microextraction automatic sampling system (Supelco Inc., Bellefonte, PA, USA) at 50 °C for 45 min. Following extraction, the SPME fiber (divinylbenzene/carboxen/polydimethylsiloxane, DVB/CAR/PDC, 2 cm, 50/30 microns) was desorbed at 250 °C for 5 min via gas chromatography, after which the samples were analyzed.

### 2.5. Determination of Volatile Components

Volatile compounds were analyzed by full two-dimensional time-of-flight mass spectrometer (GC×GC-TOF-MS, GT0620, Hexin Instrument Co., Ltd., Guangzhou, China). Instrument parameters were adapted from previously established methods [15]. The GC×GC conditions included an inlet temperature set at 230 °C, a shunt ratio of 10:1, and high-purity helium (purity > 99.9995%) as the carrier gas with a flow rate of 1.0 mL/min. The primary column was ZB-FFAP (60 m × 0.25 mm × 0.25 μm, Agilent Technologies, Santa Clara, CA, USA). The column temperature program commenced at 40 °C for 5 min, followed by a ramp to 200 °C at a rate of 3 °C/min for an additional 5 min, and finally increased to 230 °C at a rate of 10 °C/min for 5 min. The secondary column was DB-17HT (0.6 m × 0.25 mm × 0.25 μm, Agilent Technologies, Santa Clara, CA, USA), maintained at 5 °C higher than the one-dimensional column. TOF-MS conditions included a solvent delay of 3.5 min, electron impact (EI) ionization at 70 eV, an ion source temperature of 230 °C, an interface temperature of 230 °C, a detector voltage of -1700 V, a mass collection range of 45-500 *m*/*z*, and a scanning frequency of 101 spectra/s. The modulator operated with a modulation period of 5 s.

Prior to sample analysis, an n-alkane homologous series standard (C8–C40) was analyzed under identical GC×GC-TOF-MS conditions. The retention index of each detected compound was then automatically calculated by the instrument software from the n-alkane calibration data as the Kovats retention index, based on the first-dimension retention time on the ZB-FFAP column (60 m × 0.25 mm × 0.25 μm). The software-generated RI of each compound was subsequently compared with the RI of the same compound reported in the NIST 21 library [16] for the same stationary phase (FFAP-type). Only compounds meeting all of the following criteria were accepted as identification results: (i) forward and reverse spectral matching degrees greater than 800, (ii) possibility greater than 75%, and (iii) an RI deviation within ±50 between the experimental (software-calculated) value and the NIST 21 library [16] value. For semi-quantitative analysis, the relative content of each compound was expressed as the ratio of the compound’s peak area to that of the internal standard (2-octanol). This internal standard method enables comparison of the relative abundance of each compound among the samples analyzed in the same batch, but does not represent absolute concentrations.

### 2.6. Statistical Analysis

Data statistics were performed using Microsoft Excel 2021 (Office 2021, Microsoft Corporation, Redmond, WA, USA), with results expressed as mean and standard deviation (SD). Data analysis and visualization were conducted using SPSS 26 (SPSS, Chicago, IL, USA) and Origin 2024 software (MicroCal Inc., Northampton, MA, USA). Statistical significance was determined at a threshold of *p* < 0.05. For E-tongue and GC E-nose analyses, technical replicates were first averaged to obtain sample-level data. These technical replicates were used to assess analytical precision and were not treated as independent biological or production replicates. For GC×GC-TOF-MS data, the relative level of each compound was calculated as the ratio of the target peak area to the internal standard (2-octanol) peak area. Missing values were set to zero or half of the minimum detected value before log transformation. To identify compounds that differed among the analyzed samples, one-way ANOVA was performed for each compound. When normality or homogeneity of variance was not satisfied, the Kruskal–Wallis test was used. Multiple comparisons were corrected using the Benjamini–Hochberg false discovery rate (FDR) method, and compounds with *q* < 0.05 were considered differentially detected among the analyzed samples. Post hoc pairwise comparisons were performed using Tukey’s HSD test or Dunn–Bonferroni test. These statistical results are exploratory and refer only to the analyzed samples because independent fermentation or production batches were not available. For PCA and HCA, sample-level data were used. HCA was performed using Euclidean distance and Ward. D2 linkage. Heat maps were generated using z-score standardized data. Statistical significance was set at *q* < 0.05 after FDR correction. It should be emphasized that the PCA and HCA results presented in this study are exploratory. Although the two principal components accounted for more than 90% of the variance in both the E-tongue and E-nose datasets, and the groupings were consistent across three independent analytical platforms, the limited number of samples (n = 12) and the absence of independent fermentation batches mean that the observed clusters may not be stable in a larger dataset. In addition, for the high-dimensional GC×GC-TOF-MS data, the ratio of variables to samples is high, which may lead to overfitting in unsupervised analyses. To mitigate this, differential compounds were identified with FDR correction, and only compounds meeting both statistical and identification-confidence criteria were discussed. Future studies with a larger number of independent samples and batches are required to validate the robustness and generalizability of the observed discrimination and of the proposed candidate markers.

## 3. Results and Discussion

### 3.1. Taste Differences of Baijiu Samples

**Figure 1:**
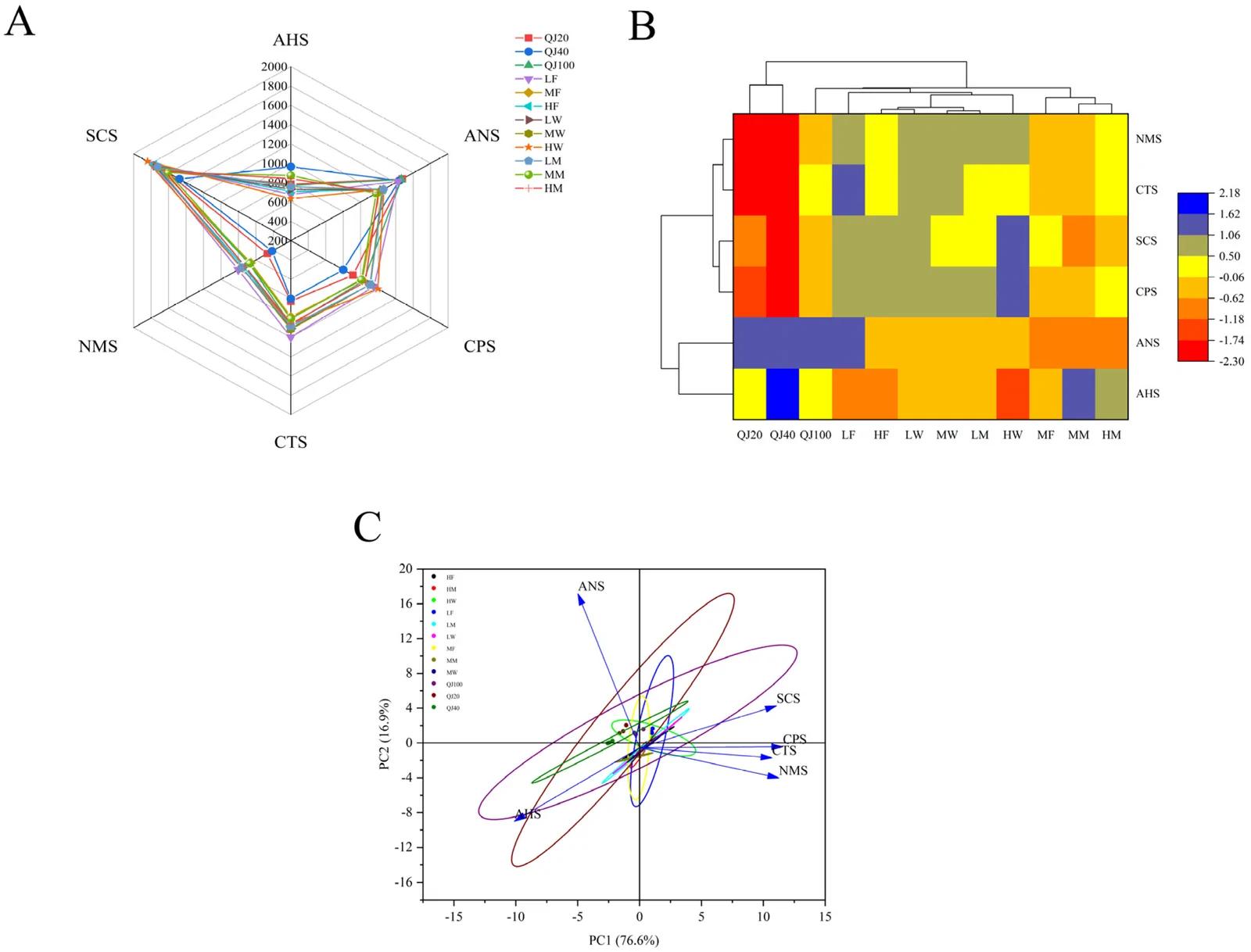
Radar plots (**A**), cluster heat map (**B**) and principal component analysis (PCA) loadings plot (**C**) of E-tongue sensor response of different Baijiu samples. Data are presented as mean ± SD (n = 3 for E-tongue).

The electronic tongue system consists of various taste sensors that are capable of detecting taste compounds in food samples [10]. The data generated by these sensors were used to identify samples through the establishment of PCA, ANN, SVM, PLSR and other classification or regression models [11]. As depicted in Figure 1A, the response values for six sensors were displayed, include those for sourness (AHS), saltiness (CTS), umami (NMS), sweetness (ANS), bitterness (SCS), and a universal sensor (CPS). There were significant differences among five taste attribute values in different flavor types of Baijiu (*p* < 0.05). Specifically, QJ20, QJ40 and QJ100 showed lower bitterness-associated sensor responses and relatively higher sweetness-associated sensor responses than the commercial reference samples. These results indicate differences in E-tongue sensor response patterns among the analyzed samples and should not be directly interpreted as human-perceived sweetness. This increased sweetness was likely due to the substantial production of glucose from the hydrolysis of carbohydrates during brewing [17]. QJ40 displayed the highest acidity attribute value, which significantly differed from the other samples (Figure 1B), potentially attributed to elevated levels of organic acids. Different levels of polysaccharides, organic acids, polyphenols and ethanol in Baijiu may have a great influence on the formation of sweetness, acidity and bitterness [13]. Additionally, the saltiness and umami values of QJ20, QJ40, and QJ100 were relatively low, indicating that quinoa Baijiu tends to have a more aromatic profile compared to commercial Baijiu.

Principal component analysis (PCA) is a statistical method that transforms the original variables into a linear combination, facilitating simplification and dimensionality reduction while retaining essential information [18]. The PCA score chart, illustrated in Figure 1C, displayed the response intensity data. Notably, the two main components of the variance accounted for 93.5% of the total variance, indicating the effectiveness of the E-tongue in effectively distinguishing between different samples of Baijiu. Visual examination of the PCA score table revealed distinct groups: the QJ20, QJ40, and QJ100 samples were clearly separated from the other samples and distributed in different quadrants. In contrast, the LF, MF, HF, LW, MW, HW, LM, MM, and HM samples were close to each other. These observations were further corroborated by Figure 1B, which delineated three distinct clusters among the samples. The combined analysis of Figure 1B,C consistently highlighted the unique E-tongue sensor response characteristics of QJ20 and QJ40, indicating a clear differentiation from the other samples. Overall, the quinoa Baijiu samples exhibited a sweeter, distinguishing them from other types of Baijiu available on the market.

### 3.2. GC E-Nose Response Differences of Baijiu Samples

**Figure 2:**
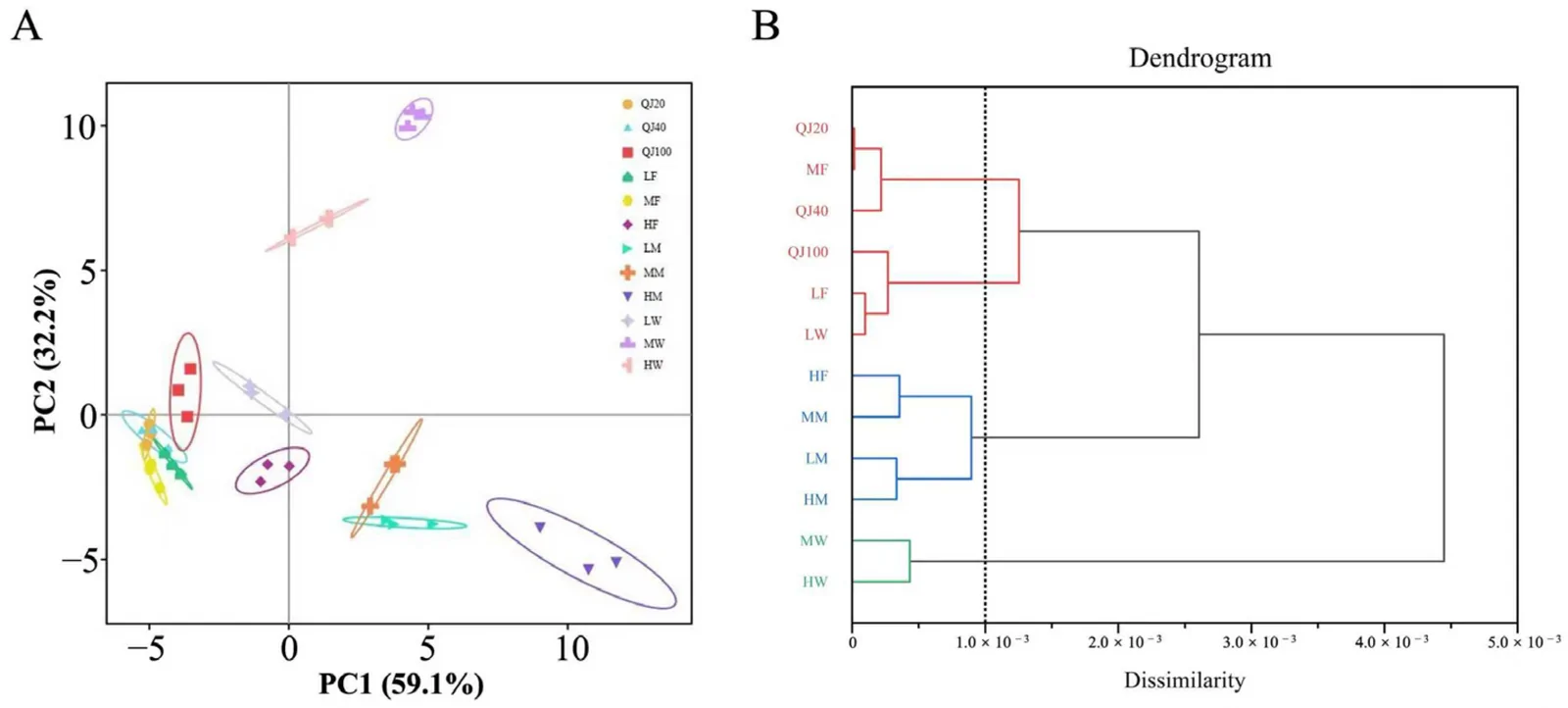
Principal component analysis (PCA) shot chart (**A**) and hierarchical cluster analysis (HCA) tree diagram (**B**) of E-nose response data of different Baijiu samples. Data are presented as mean ± SD (n = 3 for GC E-nose).

As an odor detection instrument based on the headspace phase principle, rapid GC E-nose technology captures odor components from samples using its specialized chromatographic column. This technology allows for comprehensive and swift characterization of odor properties, providing an objective reflection of the sample’s overall volatile odor characteristics [12]. As shown in Figure 2A, the PCA results of 12 Baijiu samples determined by GC E-nose were presented. Notably, the first two principal components accounted for 91.3% of the total variance, demonstrating the E-nose’s effectiveness in distinguishing between different Baijiu samples. Visual inspection of the PCA score table revealed that the three quinoa Baijiu samples were distinctly separated from other Baijiu samples, with the exception of the LF samples, and were distributed across different quadrants. Additionally, the overall flavor profiles of the three quinoa Baijiu types were similar, likely due to the presence of comparable volatile substances but varying concentrations [19].

Hierarchical cluster analysis (HCA) was employed to elucidate systematic responses and investigate similarities among different Baijiu samples [20]. As shown in Figure 2B, all Baijiu samples were classified into four distinct clusters, which aligned closely with the results of the PCA (Figure 2A). The HCA diagram indicated that QJ20, QJ40, and MF samples were positioned at the top, in proximity to QJ100 sample, while HW, MW, LM, and HM samples were located at the bottom. This clustering suggested that the differences among the various types of Baijiu were pronounced. The GC E-nose effectively distinguished the analyzed Baijiu samples based on their instrumental aroma fingerprints. This discrimination reflects sensor response patterns rather than human aroma perception. Notably, the instrumental aroma fingerprints of the three quinoa Baijiu samples were like those of Fenjiu samples. These findings further corroborated the results obtained from the PCA.

### 3.3. Differences in Volatile Components of Baijiu Samples

**Figure 3:**
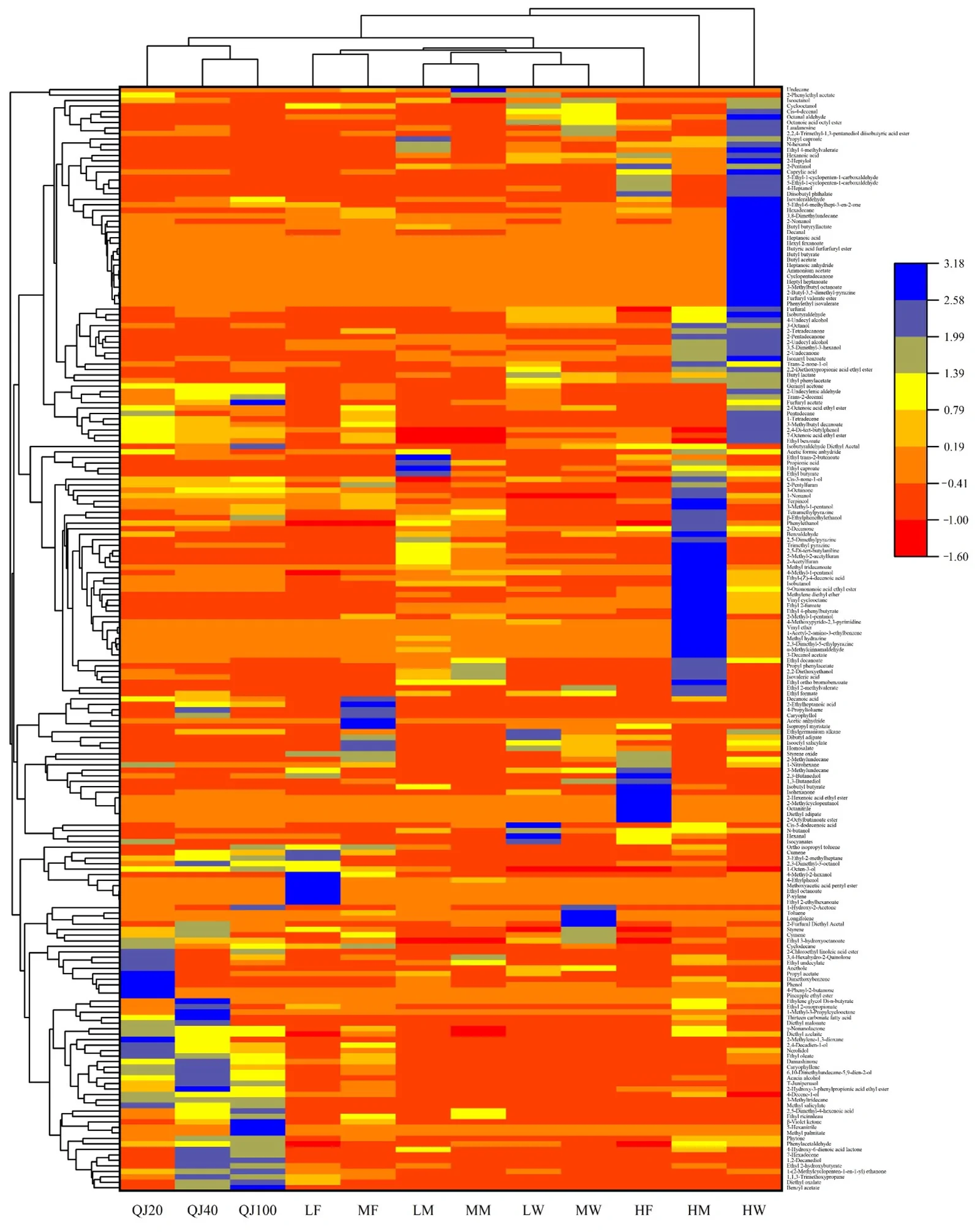
Heat map and HCA clustering results of 201 aroma compounds with a significant difference in 12 samples of Baijiu (*p* < 0.05).

**Figure 4:**
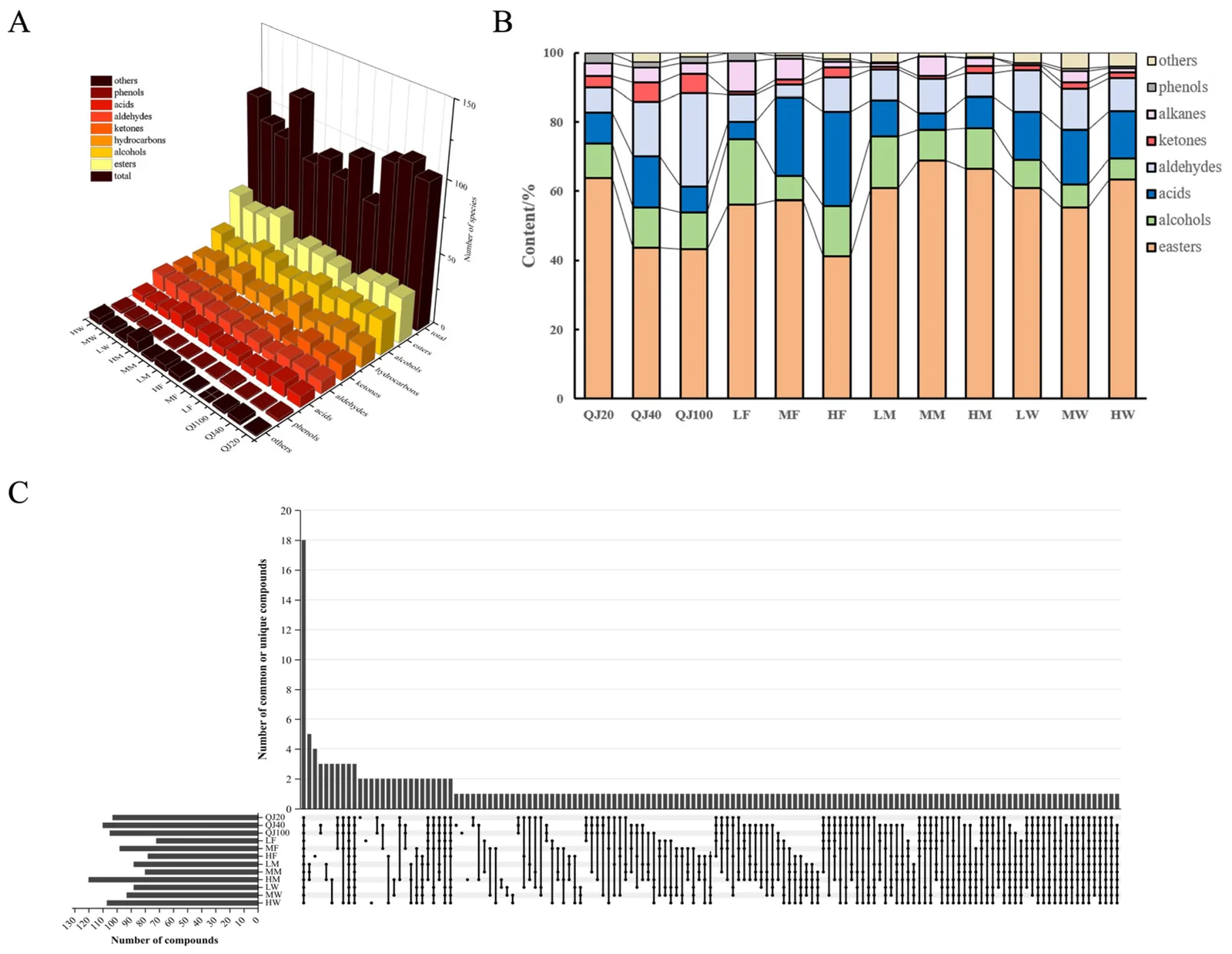
The species statistical diagram (**A**), the content statistical diagram (**B**) and upset diagram (**C**) of the volatile components of different Baijiu samples. Data are presented as mean ± SD (n = 3 for GC×GC-TOF-MS).

A total of 201 compounds were successfully identified within the samples using GC×GC-TOF-MS. These compounds comprised 70 esters, 37 alcohols, 12 acids, 16 aldehydes, 16 ketones, 36 hydrocarbons, 3 phenols, and 11 other compounds, as illustrated in Figure 3. Notably, Figure 4A revealed the quantities of volatile flavor substances in each Baijiu sample. Specifically, QJ20, QJ40, QJ100, LF, MF, HF, LW, MW, HW, LM, MM, and HM contained 103, 110, 105, 72, 98, 79, 88, 93, 108, 88, 80, and 120 compounds, respectively. This distinction underscores the remarkable diversity in their characteristic profiles. Figure 4C further nuanced revealed the characteristic volatile flavor compounds present in the Baijiu samples. Each Baijiu sample had a group of candidate volatile markers, which constituted their unique characteristics. In summary, the differences in the types and quantities of volatile flavor compounds among Baijiu varieties can be explained by several factors. First, raw materials provide the substrate basis: the substitution of quinoa introduced distinct starch, protein and lipid compositions. Second, production techniques play a decisive role: the ceramic-jar fermentation and short-cycle “steaming twice and clearing” process of light aroma Baijiu may account for the relatively small number of volatile compounds detected in LF. Third, microbial communities mediate these processes, since esters, alcohols and acids are primarily synthesized by yeasts and lactic acid bacteria during fermentation, and variations in pit mud or Daqu microbiota among the aroma types contribute to their distinct flavor profiles. Finally, storage conditions affect aging-related transformations. These interpretations are consistent with previous studies [21,22,23]. It should also be noted that comparisons are valid among samples of the same batch but should not be interpreted as absolute concentrations. Compound-specific calibration curves and response factors will be established in future studies to enable absolute quantification.

#### 3.3.1. Esters

Among all flavor compounds, esters accounted for over 60% of the total relative peak area of flavor substances, and many esters exhibited low odor thresholds, indicating their significant contribution to aroma [24]. A total of 70 esters were identified in 12 liquor samples, of which 32, 34, 31, 22, 29, 31, 37, 36, 43, 32, 25 and 43 were identified in QJ20, QJ40, QJ100, LF, MF, HF, LW, MW, HW, LM, MM and HM samples, respectively. Among these identified esters, ethyl esters accounted for the largest proportion, aligning with findings from previous studies [25]. Within the three types of quinoa Baijiu, the most abundant esters were ethyl butyrate, ethyl caproate, ethyl caprylate, ethyl undecanoate, ethyl oleate, and propyl acetate, which could serve as predominant esters and may serve as candidate chemical markers detected in the analyzed quinoa Baijiu samples. Ethyl caproate has been reported to impart a pineapple-like flavor; however, its actual sensory contribution in quinoa Baijiu was not determined in this study [26]. Similarly, ethyl butyrate had a fragrance reminiscent of pineapple and banana, and its appropriate concentration can enhance the freshness and cleanliness of Baijiu [27]. In addition, compared to commercially available Baijiu, four ester compounds were only identified in the quinoa Baijiu samples: methyl palmitate, 2-chloroethylidene oleate, ethyl pineapple, and phenyl methyl acetate. These compounds contributed to the distinct flavor characteristics of quinoa Baijiu. Collectively, these esters imparted a unique aroma, sweet, and harmonious taste, which aligned with the findings presented in the previous section. Figure 4B underscored the diversity of ester peak-area proportions, ranging from 41% to 69%. The microbial synthesis of esters was generally recognized as the primary pathway for ester formation in Baijiu [28]. Variations in yeast strains across different types of Baijiu may result in differences in total ester content [29].

#### 3.3.2. Alcohols

Alcoholic compounds also play a significant role in the aromatic profile of Baijiu, primarily produced from sugars in raw materials under aerobic conditions or through the reduction of aldehydes by yeast and other microorganisms [30]. A total of 37 alcohol compounds were identified across 12 Baijiu samples. The types and concentrations of alcohol compounds were similar among the three varieties of quinoa Baijiu. Notably, quinoa Baijiu exhibited higher relative alcohol abundance compared to strong aroma Baijiu and sauce aroma Baijiu. Furthermore, all Baijiu samples contained alcohol compounds with high relative content, including phenylethanol, n-butanol, isobutanol, n-hexyl alcohol, 1-nonyl alcohol, and 4-decene-1-alcohol, which collectively accounted for over 90% of the total alcohol content. These alcohols contributed to floral, resinous, green, and fresh aromas [31]. Additionally, 1,2-sebacediol and acacia alcohol were exclusively detected in quinoa Baijiu, suggesting their potential as discriminative volatile markers; their sensory contribution remains to be determined.

#### 3.3.3. Acids

Acids are essential components in Baijiu, contributing to flavors reminiscent of fruit, cheese, fat, and rancidity. These acids are primarily produced by yeast and lactic acid bacteria [32]. The appropriate concentration of acids is crucial for achieving balance and harmony in the overall flavor profile of Baijiu, as excessive acidity may adversely affect its aroma [26]. A total of 12 acid compounds were identified across all Baijiu samples, with common and predominant acids including caproic acid, caprylic acid, capric acid, and heptanoic acid, highlighting the similarities in acid composition and content. Caproic acid, a saturated fatty acid consisting of six carbon atoms and one carboxyl group, can be transformed into valuable compounds such as ethyl caproate and hexyl alcohol through esterification and hydrogenation [33]. Notably, the highest relative contents of capric acid and caprylic acid were observed in the QJ20 Baijiu sample, while the QJ40 Baijiu sample exhibited the highest relative level of 2-ethyl heptanoic acid. Furthermore, 2,5-dimethyl-4-hexenoic acid was exclusively detected in quinoa Baijiu, indicating it as a candidate chemical marker for the analyzed quinoa Baijiu samples.

#### 3.3.4. Aldehydes and Ketones

Ketones and aldehydes significantly influence the aroma and aging characteristics of Baijiu, primarily serving a coordinating role in its overall flavor profile [34]. The formation of these compounds during the fermentation process predominantly arises from yeast metabolism and the oxidation of alcohol [35]. In the analyzed samples, a total of 16 aldehydes and 16 ketones were identified. Notably, common and abundant aldehydes included acetaldehyde, isovaleraldehyde, furfural, hexanal, phytoketone, 2-decanone, and 2-undecanone, with acetaldehyde and furfural being particularly prevalent. Acetaldehyde contributes positively to the aroma of Baijiu, while furfural is generated from the poly-pentose acetals found in wheat husks and other raw materials. As the number of fermentation rounds increases and the consumption of wheat husks rises, the concentration of furfural will also increase correspondingly [36]. A comparative analysis of three quinoa Baijiu samples revealed that benzaldehyde was exclusively detected in the QJ20, imparting notes of bitter almond, cherry, and nut. The QJ100 exhibited the highest levels of isovaleraldehyde and furfural, which can be closely attributed to the substantial addition of quinoa during brewing. Notably, β-violone and damasone were exclusively identified in quinoa Baijiu and light aroma Baijiu, and β-violone has been reported to possess cypress and raspberry like aromas. However, its aroma contribution in the present samples was not validated.

#### 3.3.5. Other Flavor Compounds

The flavor compounds present in various types of Baijiu also encompassed phenols, alkanes, pyrazines, furans, terpenes, and others, each contributing uniquely to the overall flavor profile. For instance, all three varieties of quinoa Baijiu exhibited high relative level of caryophyllene, which has been reported to impart woody, clove, and mint aromas, along with notable health benefits [37]. Additionally, a low concentration of paracymene was detected in both quinoa Baijiu and light aroma Baijiu, and the increase in the content of the compound played a role in promoting the presentation of sweet and woody flavors in vintage Baijiu. The level of 2,4-di-tert-butylphenol in quinoa Baijiu was particularly high, contributing a distinct carbolic flavor. In addition, phenolic compounds are well-known contributors to bitterness and astringency in alcoholic beverages. In this study, only three phenolic compounds were detected in the volatile fraction by GC×GC-TOF-MS, among which 2,4-di-tert-butylphenol was present at a particularly high level in quinoa Baijiu. Therefore, the low bitterness values of quinoa Baijiu may be partly attributed to its relatively low content of bitter phenolics, together with the masking effect of its high sweetness and abundant esters. It should be noted that the total phenolic composition was not quantified in this study, which we acknowledge as a direction for future work. Furan and pyrazine compounds arise from Maillard reactions occurring during high-temperature accumulation, fermentation, and distillation, serving as critical aroma and functional components in Baijiu [38]. For instance, the presence of 2-amyl furan in quinoa Baijiu imparted a green, vegetal flavor. It was noteworthy that while pyrazine compounds were abundant in sauce aroma Baijiu, and they were either absent or present in minimal quantities in other samples. Furthermore, the flavor profile of quinoa Baijiu was closely linked to the specific microorganisms involved in the fermentation process [39]. To gain a deeper understanding of the flavor characteristics of quinoa Baijiu and differences among various Baijiu types, further research into the interactions between fermentation agents and fermentation process is essential.

The distinct volatile profiles of the Baijiu samples can be largely explained by their production technologies. Light aroma Baijiu (Fenjiu) is characterized by ceramic-jar fermentation and the “steaming twice and clearing” process. This relatively clean and short-cycle process may explain why the LF sample contained the fewest volatile compounds among all samples, and why β-violone and damasone were shared between light aroma and quinoa Baijiu, as quinoa Baijiu was also brewed with a light-aroma-type process in Shanxi. Regarding the quinoa Baijiu samples, the substitution of quinoa for traditional grains introduces a distinct raw-material basis, which plausibly accounts for the characteristic compounds exclusively detected in quinoa Baijiu (e.g., methyl palmitate, 1,2-sebacediol, acacia alcohol and 2,5-dimethyl-4-hexenoic acid), as well as the elevated furfural and isovaleraldehyde in QJ100 with increasing quinoa proportion. It should be noted that detailed process parameters (e.g., fermentation temperature, duration and Daqu type) were not disclosed by the producer. Elucidating the exact links between these parameters and the flavor fingerprints will be the focus of our future work.

## 4. Conclusions

In this study, we analyzed the differences and similarities between quinoa Baijiu with varying levels of quinoa addition and three classic flavor types of Baijiu: light aroma, sauce aroma, and strong aroma. Utilizing HS-SPME in conjunction with GC×GC-TOF-MS, as well as GC E-nose and E-tongue techniques, we identified several key characteristics of quinoa Baijiu, to our knowledge, the first instrumental flavor characterization of quinoa Baijiu. These preliminary findings may provide a reference for future, larger-scale studies on quinoa Baijiu flavor quality. Firstly, E-tongue and GC E-nose analyses provided instrumental fingerprints that discriminated the analyzed quinoa Baijiu samples from the commercial reference samples. The quinoa Baijiu samples showed higher sweetness-associated and lower bitterness-associated E-tongue responses, and their E-nose aroma fingerprints were similar to those of light-aroma Baijiu. These results are instrument-based and do not constitute human sensory characterization. Secondly, E-nose analysis revealed clear distinctions among the different types of Baijiu. However, the instrumental aroma fingerprints of quinoa Baijiu and Fenjiu exhibited notable similarities. Thirdly, key ester compounds prevalent in quinoa Baijiu included propyl acetate, ethyl undecanoate, ethyl oleate, and diethyl azelate. Other significant compounds identified at relatively higher abundance were 1,2-sebacediol, acanthyl alcohol, 2-ethyl heptanoic acid, 2,5-dimethyl-4-hexenoic acid, benzaldehyde, 1-methyl-3-propylcycloctane, 3-methyl-tridecane, 7-hexadecene, and caryophyllene. Importantly, among the samples analyzed, the volatile flavor components in quinoa Baijiu were distinctly different from those found in traditional Baijiu, which supported discrimination among the analyzed samples. However, since 0% quinoa control samples produced under the same conditions were not included, these differences could not be completely attributed to the addition of quinoa. Cluster analysis results indicated that quinoa Baijiu can be effectively differentiated from other types of Baijiu, highlighting its unique flavor profile. Overall, while limited by the sample size, these findings provide an initial chemical basis for the discrimination of quinoa Baijiu and for further studies on its flavor quality.

## Data Availability

Data will be made available on request.
